# Moving motives: How past and present strategy influence the market

**DOI:** 10.1371/journal.pone.0259660

**Published:** 2021-12-08

**Authors:** Rick H. L. Aalbers, Killian McCarthy, Menno Huisman, Jonas Roettger

**Affiliations:** 1 Strategy and Innovation, Centre for Organization Restructuring, Radboud University Institute for Management Research, Nijmegen, The Netherlands; 2 Strategy and Innovation Management, Centre for Organization Restructuring, University of Groningen Faculty of Economics and Business, Groningen, The Netherlands; 3 SeederDeBoer, Amsterdam, The Netherlands; 4 Centre for Organization Restructuring, Radboud University Institute for Management Research, Nijmegen, The Netherlands; Bucharest University of Economic Studies, ROMANIA

## Abstract

We investigate the market’s reactions to serial acquirers that switch strategy. We collect data on 204 serial acquirers in four high tech industries, and use March’s explore-exploit framework, to classify these firms’ 1,415 acquisitions. We then distinguish, for example, exploration-based acquisitions, conducted after a series of exploitation-based acquisitions. Our results suggest that the market takes a portfolio perspective when reacting to an acquisition. In support of the ambidexterity literature, we show that the market responds positively to a switch from one type of strategy to another. Zooming in on the direction of the shift, we find that the market responds more positively to a switch towards exploration after exploitation, compared with the alternative. In so doing, we contribute to the literature on acquisition motives, by showing that prior announcements matter in explaining market reactions, and we contribute to the literature on ambidexterity, by showing that the market favours firms that oscillate between exploration and exploitation.

## 1. Introduction

Research often considers the performance of a single acquisition, or the effects of a single acquisition on the performance of the acquiring firm (e.g., [1]). This shows that the acquisition motive matters. For example, [2] finds that acquisitions aimed at exploitation outperform acquisitions aimed at exploration.

Firms often, however, make multiple acquisitions, back-to-back [3–5]. And research recognizes that firms must both explore and exploit to sustain their competitiveness [5]. There is evidence too to suggest that firms use some acquisitions to explore and others to exploit. For example, in 2020, *Alphabet*, the mother company of *Google*, bought *Dataform*, to exploit its data analytics capabilities, and in 2021, it bought *Fitbit*, to explore the wearables market.

This raises a number of interesting questions. For example, do investors take the ’portfolio’ of acquisitions into account when reacting to an announcement of an acquisition? Does the market react to the switch in strategy from exploration to exploitation? And if it does, does the market prefer a switch to exploration or exploitation?

Existing research—in both strategy and in finance—suggests that these things should matter (e.g., [2,6,7]). This shows that markets incorporate a range of new information when updating the expected future value of a firm [8–10]. For example, [2] shows that the market positively updates the value of a firm that announces a low-risk acquisition, aimed at cutting-cost, and it reacts more negatively to the announcement of a high-risk acquisition aimed at exploring new technologies. Surprisingly, only a few scholars have investigated the way in which the market reacts to the announcement of individual acquisition motives, and none, to the best of our knowledge, has considered the way in which the market reacts to a ’series’ of announced motives.

Based on recent contributions to the literature (e.g., [2,11,12]) we argue that the market–which is by definition risk-averse–will take the serial acquirer’s ’portfolio’ of acquisitions into account when reacting to the announcement of the focal acquisition. Building on the literature on ambidexterity [13], which suggests that the firm should both explore and exploit, we suggest that the market will value a ’switch’ in strategy. What is more, we suggest that the market will prefer a switch to exploration, after a sequence of exploitative acquisitions, compared to the switch to exploitation, after a sequence of explorative acquisitions. We argue that this is the case because in an innovation context, for example, the switch to exploration signals that the firm is ready to engage in high-quality innovation aimed at long term performance [14] and, after a series of exploitative acquisitions, it has the slack resources necessary to fund these innovations too [15].

We test our hypotheses using a sample of 204 serial acquirers in 4 high tech sectors that performed 1,415 acquisitions in the period 2000–2016. Our results suggest that the market reacts positively to a switch in strategy and that the type and direction of the switch matters.

In so doing, we provide a longitudinal view onto a firm’s use of acquisition, and make contributions to a number of literatures. For example, we extend the innovation literature, on the one hand, by applying March’s [5] framework of explore-exploit to the topic of serial acquisitions, and signaling theory, on the other, by introducing the switch as a signal which affects market reactions. We contribute to the work on acquisitions (e.g., [1]), by exploring performance effects, to work on acquisition motives (e.g., [1,2]), by demonstrating that the series and sequence of motives matters, and we contribute to the work on serial acquirers, by incorporating an acquirer’s previous acquisition strategy, in response to calls to do so (e.g. [16,17]). Our key contribution, however, is to the literature on ambidexterity. We show that firms can and should use acquisitions to explore and to exploit, and show that the market rewards firms for systematically oscillating back and forth between exploration and exploitation.

## 2. Theoretical background

### 2.1 Serial acquirers

The literature on serial acquirers has explored the question of why firms become serial acquirers. On the individual level, traits such as narcissism, overconfidence, and extraversion have been put forward and found to have an effect on acquisitiveness [18–20]. On the organizational level, the occurrence of a high frequency of acquisitions is argued to be the consequence of disappointing firm performance and a "desperation to grow" [21]. Finally, at a more macroeconomic level, changes in the firm’s environment, such as regulatory and economic shocks [22], have been put forward to explain serial acquisitions.

### 2.2 Acquisition motives

There is a growing body of work on acquisition motives in finance (e.g., [23]) and strategy (e.g., [2,24]), which investigates acquisition motives. Most of this either infers motives from post-acquisition firm performance (e.g., [25]) or infers motives from acquirer industry (e.g., [26]) or target type (e.g., [27]). Only a few studies have empirically explored the link between the declared pre-acquisition announced motive to the firm’s post-acquisition performance (e.g., [28]). For example, [2] uses press releases to identify and categorize announced acquisition motives and then considers the way in which the market reacts to the acquisition with different motives. Despite the calls to delve deeper into acquisition motives (e.g. [29]), there remains much work to be done. For example, there is no work, to the best of our knowledge, that has considered the performance consequences of announced motives in relation to serial acquirers, leaving our understanding of acquisition patterns underdeveloped to date.

### 2.3 Classifying acquisition motives

[30] introduced the concepts of exploration and exploitation. Since then, the framework has become an inexhaustible source of research within various literature streams in management. Today, it is one of the most widely used subcategorizations in strategy.

Exploration is about "search, variation, risk taking, experimentation, play, flexibility, discovery, [and] innovation" ([30] p.171). In the context of acquisitions, [2] and [31] suggest that exploration is about expanding into new products and services, new industries, and new geographic regions. It is about learning to create new products, services, and markets or about accelerating innovation. It is about accessing intellectual property, patents, knowledge or technology, to enable the firm to explore new technological domains. It is about ’newness’, which is a higher risk [23].

Exploitation, by contrast, is a short-term and certain strategy that involves ’refinement, choice, production, efficiency, selection, implementation, [and] execution’ ([30] p. 71). In the context of acquisitions, [31] and [2] suggests that exploitation is about improving your financial position or about reducing your tax exposure. It is about building economies of scale and scope, cutting costs, or vertically integrating to improve supply chains. It is about strengthening the core business, building size, and scale to win in your current market. Exploitation is about the ’sameness’. It is immediate, more readily estimated, and therefore a lower risk than exploration [23].

Neither strategy is superior, and firms must explore and exploit in order to maintain their competitiveness [2]. Exploitation creates new knowledge, for example, by gradually adapting existing routines and capabilities already present within the organization [32,33]. As a result, because exploitation allows the firm to carry out its activities more efficiently, exploitation often generates slack resources too [34,35]. Exploration, by contrast, brings the sorts of new knowledge, new knowledge domains, and knowledge production routines, which are necessary to develop breakthrough innovation [36–39]. Exploration increases the firm’s absorptive capacity, improving its abilities to scan external information and utilize it for commercial benefits [32,40]. As a result, exploration increases its adaptability to environmental changes [41].

Even though exploration and exploitation can increase complexity when performed simultaneously, both innovation types are fundamental for firm performance. Exploitative innovation, for example, leads to incremental innovation necessary to maintain a continuous stream of revenues in the short term, while exploratory innovation leads to the breakthrough innovations that create new products, markets, and consumers, which creates a continuous stream of revenues in the long term [42]. In fact, an overinvestment of resources in exploitation can result in a depletion of opportunities, whereas too much emphasis on exploration leaves an organization with a surplus of underdeveloped ideas and untapped opportunities [30]. Therefore, it is essential for firm performance to balance innovative activities between exploratory and exploitative innovation [30].

### 2.4 Acquisition motives and market reactions

Signaling theory [43,44] is used in situations where information is both incomplete and asymmetrically distributed [43–47]. The literature on signaling theory considers how ’senders’ send ’signals’ to ’receivers’. In a strategy context, the signal ’sender’ is typically the firm or the manager, the ’signal’ is an activity or attribute that, by design or accident, conveys information that alters the receiver’s beliefs or behavior in terms of the firm, and the ’receiver’ is often the stock market [48,49]. The efficient-markets hypothesis [50] suggests then that the market will incorporate all such signals, that might affect the future value of the firm into its current market price [51].

In the context of an acquisition, the acquiring firms is the signal sender, the signal is the announced acquisition to the market, which conveys information that alters the markets beliefs about the future value of the firm, and the market is the receiver [48,49]. Through the acquisition announcements, the firm attempts to outline how this acquisition will increase firm performance [52,53]. This study views these signals as an ’lever’ that affects market response. Given that firms often perform multiple acquisitions within a short period to achieve their desired outcomes [17], we build on the suggestion that instead of evaluating and analyzing ’an’ acquisition in isolation, we should instead analyze the firm’s ’acquisition program’ (e.g. [5,54]). As serial acquirers perform various acquisitions in a short time window, we suggest that it is credible that investors will be aware of previous events and previously deployed strategies. Therefore, we develop two hypotheses regarding serial acquirer acquisition announcement and market responses to them.

## 3. Hypotheses development

### 3.1 Market reaction to switching

To survive, a firm must explore–with one eye on the future–but it also needs to exploit—with the other eye on the present [30]. A firm that does this is termed an ’ambidextrous’ firm [55]. Ambidexterity is desirable because it exposes the firm to revolutionary and evolutionary sources of change [56].

The literature describes a number of ways in which a firm can become ambidextrous. Structurally, for example, ambidexterity could be achieved by creating separate exploration and exploitation units within the firm, each outfitted with different people, structures, processes, and cultures [13]. An alternative is to ’sequentially switch’ (Brown and Eisenhardt, 1997), or by ’oscillating back and forth between periods of exploitation and exploration’ ([57], p. 327). The critical points, however, is that the firm does both, and not one at the cost of the other: to focus on both short-term (exploitation) and long-term (exploration) performance, without neglecting the need for the other one [7,58].

Following on the suggestion that when investors understand the aim of and have confidence in the firm’s strategy, they will translate this into positive market reactions (e.g., [2,6,12]), we argue that a ’switch’ in strategy will be welcomed by the market. Specifically, we suggest that the market will react positively to a switch towards exploration after a series of exploitation, and to a switch towards exploitation after a series of exploration. In either case, we suggest the switch will be seen as a value-creating step towards building a more balanced position. Thus:

*Hypothesis 1*: *The market will respond positively to a switch in acquisition strategy by a serial acquirer*.

### 3.2 Market reaction to the direction of the switch

There are clear reasons why the market might prefer exploitation over exploration. For example, from a short-term financial performance perspective, research suggests that exploitation is useful for building up slack resources [35]. Also, from a valuation perspective, research shows that the market is less able to value exploratory strategies, as a larger knowledge gap exists between managers and the capital market [59]. Incidentally, [60] argues that, because of this gap, firms pursuing exploratory endeavors have a higher chance of stock price crashes due to the publication of ’bad news’.

There are clear reasons too, however, to suggest that the market will react more positively to a switch towards exploration after a series of exploitation, compared to a switch towards exploitation after a series of exploration. First, in an innovation context, for example, innovations resulting from exploratory activities provide better results than innovations created by exploitative innovation [36,61]. A switch to exploration, after a series of exploitation, could therefore signal that the firm is searching for high quality, radical innovation aimed at long-term performance [14]. Secondly, a sequence of exploitative acquisitions can provide firms with significant slack resources [15,62]. These can be used to enable the firm to successfully take the ’leap into the unknown’ in the pursuit of exploratory innovation [63]. A switch to exploration, after a series of exploitation, could therefore signal that the firm is in procession of the necessary resources to fund and maintain high quality, radical innovation while absorbing the setbacks that these entail. [64].

Consequently, and following on the suggestion that when investors understand the aim of and have confidence in acquirers strategy they will translate this into positive financial results on the stock market (e.g., [2,6,12]), we argue that a switch towards exploration will be welcomed by the market. Specifically, we suggest that the market will react more positively to a switch towards exploration after a series of exploitation, and less positively to a switch towards exploitation after a series of exploration. Taken together, therefore, we propose:

*Hypothesis 2*: *The market will respond more positively to a serial acquirer that switches from exploitation to exploration to one that switches from exploration to exploitation*

## 4. Methods

### 4.1 Setting

We test our hypotheses using a sample of acquisitions from four high-tech sectors. We make use of this setting because knowledge in these industries quickly becomes trivial, and acquisitions are an important instrument to increase and renew knowledge in order to remain a competitive advantage [65]. We define the high-tech industries as the pharmaceuticals (SIC-code 283), computers (SIC-code 357), electronics and communications (SIC-code 36), and aerospace and defence (SIC-codes 372 and 376) industries.

### 4.2 Sample

We collect our data from *Thomson Reuters SDC*. We filtered the data to include: (1) all acquisitions; (2) involving stock-listed companies; (3) announced within the period 01/01/2001 to 01/01/2016; (4) with a deal value of >$10 million, (5) in which 100% of the target firm was acquired by the acquiring firm, and (6) both firms are active in the high-tech industries. We exclude recapitalizations, self-tenders, and repurchases, within-firm restructuring, and any acquisitions in which the acquiring and target firm were are both owned by the same parent. Doing so creates an initial sample of 3,186 acquisitions. Next, we apply the condition that the acquisitions should be completed by a serial acquirer. We follow Laamanen and Keil (2008) and define a serial acquirer as a firm that makes at least four acquisitions within ten years. We eliminate all other observations. Doing so, we create a final sample of 204 serial acquirers, who together performed a total of 1,415 acquisitions.

### 4.3 Dependent

We use an event study methodology to evaluate the market’s reaction to the announcement of an acquisition [12,66].

We estimate the event study and calculate abnormal returns using a ’market model’ [67]. Abnormal returns are the returns to the firm in excess of what was expected and are attributable to the event in question. The return of firm i on day t:

$$R_{it}=\alpha_{i}+\beta_{i}R_{mt}+\varepsilon_{it}$$ Eq (1)

where *R*_*it*_ is the return to firm i on day t, *R*_*mt*_ is the return of the ’reference market’ on which the firm is listed, on day t, to identify the portion of the return that is related to variation in the market’s return, *α*_*i*_ is the intercept term, *β*_*i*_ is the systematic risk of stock i, and ∑_it_ is the error term, with E(∑_it_) = 0. We estimate abnormal returns to the firm as:

$${AR}_{it}=R_{it}-(a_{i}+b_{i}R_{mt})$$ Eq (2)

where *a*_*i*_ and *b*_*i*_ are the ordinary least squares (OLS) parameter estimates obtained from the regression of *R*_*it*_ on *R*_*mt*_ over the ’estimation window’ (T). AR_it_ is the return to the firm, above what is expected as a ’normal’ return. The sum of the abnormal returns (ARs), over a predefined ’event window’, is referred to as the cumulative abnormal returns (CARs).

We use COMPUSTAT to identify the primary, or reference market, on which the firm is listed and to retrieve the firm- and market-level data necessary to estimate CARs; in total, we make use of 106 reference markets. We use a 260-day estimation window and a three-day event window. We follow precedent and measure abnormal returns to the acquirer in the period from one day before (-1), to one day after (+1) the announcement (e.g., [12,68]). We use a short window to minimize the inclusion of confounding effects that can lead to false inferences about the significance of the event [69]. We start with one day before the announcement in order to capture the effect of rumors and leaks on the share price [70].

### 4.4 Independent

#### 4.4.1 Acquisition motives

There are many ways to identify acquisition motives. For example, conference calls, SEC filings, in the US, or managerial surveys can be used. We make use of the press release announcing the deal because press releases are: (1) credible, (2) static public statements, which are designed (3) to communicate motive, and (4) to provoke a response.

By contrast, SEC filings are public statements, whose primary purpose is regulatory compliance. Conference calls are private conversations, which are guarded because managers want to avoid provoking a market reaction. And whereas memories change over time, making it difficult to survey managers about what the purpose might have been, press releases are static documents of historical record. What is more, press releases are credible records because all public firms are required to describe their acquisitions, and managers who make ’false and misleading statements’ can, in the US, for example, be jailed for up to 20 years.

We identify the motive underlying each acquisition, and we classify these following [2]. They categorize these as exploitative acquisitions. Specifically, we identify seven acquisition distinct motives: (1) technological acquisition, aimed at acquiring technology; (2) expansionary acquisitions, aimed at entering new products or markets; and (3) learning acquisitions, aimed at accelerating innovation. These they categorize as explorative. They then identify: (4) Financial acquisitions, motivated by, for example, tax considerations; (5) economic acquisitions, motivated by, for example, economies of scale; (6) strategic acquisitions, motivated by, for example, access to distribution channels; and (7) market share acquisitions, aimed at building size in a specific market. Then we classified these motives using [30] exploration-exploitation categorization. We are able to do so for 1,366 of the acquisitions in our sample. We label the rest as unknown motives.

To ensure correct coding, we followed recent research procedures [71]. We recruited two research assistants and trained them separately. We provided them with a set of motives and a list of example terms. We asked them to classify each acquisition by motive. They were not permitted to confer with each other, we did not explain the purpose of the study, and we did not introduce them to the exploration-exploitation framework. This way, we ensured that the classification procedure was applied objectively.

We cross-checked the quality of the data, which was produced in two ways. Firstly, we evaluated the inter-rater reliability between the two researchers, which was 86%. This rate is significantly higher than the recommended threshold of 70% proposed by [72]. We recorded the cases over which there was disagreement between the two coders. Second, we randomly selected 100 US acquisitions from the sample and, following [2], coded their motives using SEC filings. In 100% of cases, we found that the same acquisition was coded in the same way, irrespective of whether the announcement of the filing was used.

#### 4.4.2 Acquisition strategy and strategy switches

We compare the motive in the current acquisition with the motives for the firm’s previous three acquisitions, in order to identify a switch in strategy. To do so, we first identified the firm’s strategy. We did this by counting the number of exploitative and exploratory motives per acquisition. We counted the total number of motives per event and the ratio of exploratory innovation strategy motives compared to exploitative motives. We then estimated the firm’s serial acquisition strategy (*SAstrategy*) as the ratio of exploratory motives to the total motives identified in the previous three acquisitions. *SAstrategy* varies between 0 and 1, where a 1 would mean that the last three acquisitions that the firm announced only had exploratory motives, whereas a 0 would mean that the last three acquisitions did not have a single exploratory motive.

We zoom in our analysis on acquirers with a ’high’ level of focus in their previous acquisitions, meaning that the strategy followed in the previous three acquisitions leaned towards an exploratory or an exploitative strategy. An acquirer with a ’high’ level of focus is one with an aggregated exploratory strategy outside the threshold of 30–70%. This means that less than 30% or more than 70% of the identified motives, in their previous three acquisitions, had exploratory motives. For robustness checking purposes, we relax this threshold to the 35–65% range, to identify acquirers with a ’medium’ level of focus, and relax it further, to the 40–60% range to identify acquirers with a ’low’ level of focus.

Finally, we identify a strategy switch when the strategy in the focal acquisition differs significantly from the aggregate innovation strategy followed in the previous three acquisitions. We identify strategy switches that are: (1) 50%; (2) 40% and; (3) 30% different when compared to the strategy followed in previous acquisitions. As an illustration, suppose, for example, that Firm A had a serial acquisition strategy (*SAstrategy*) of 75%, meaning that 75% of the motives announced in relation to its last three acquisitions were exploratory motives. Suppose further that the strategy in the focal acquisition is 33%, meaning that 33% of the motives announced in relation to that acquisition are exploratory motives. This would be classified as a strategy switch, when we use the 30% and 40% thresholds; the switch from 75% exploratory to 33% is a 42% switch. However, if we apply the more conservative definition, and require a 50% difference, then the focal acquisition would not be qualified as a strategy switch.

### 4.5 Control variables

We control for a number of factors known to affect market reactions [73]. Specifically, we control for: (1) motive count, defined as the total number of motives announced per acquisition; (2) the acquirer’s financial slack, measured as the normalized function of the acquiring firm’s operating cash flow over its total assets in the year before the acquisition; (3), the percent of cash used in the deal; (4) the value of the acquisition expressed in US$ millions; (5) the firms prior performance, defined as its return on assets (ROA) in the year preceding the focal acquisition; (6) the size of the acquirer; (7) the geographic distance between the acquiring and target firm in kilometers; (8) the relatedness between both firms, in terms of 3-digit SIC code; (9) international deals (1 = international, 0 = domestic). Finally, we create *Acquisition Year Dummies* to control for year-specific effects and create *Acquirer’s Industry Dummies* to control for the acquirer’s industry specific effects. We inspect the distributions of each variables and use the Shapiro-Wilk Test to test for normality. We employ logs of any variables (specifically *Acquirer Size*, *Deal Value*, and *Financial Slack*) that were not normally distributed. All data to create these control variables was obtained either through the *SDC* or *Datastream* databases

### 4.6 Estimation model

In order to calculate differences in the stock market reactions as a result of the acquisition announcements of the serial acquirer, we use the following model:

$${CAR}_{it}=\beta_{0}+\beta_{1}{StrategySwitch}_{it}+\beta_{j}{Controls}_{it}+\varepsilon_{it}$$ Eq (3)

In this: *CAR*_*it*_ is the returns to firm i on an acquisition at time t. *β*_1_*StrategySwitch*_*it*_ is either: (1) a dummy variable which identifies a switch from exploration to exploitation, or (2) a dummy variable which identify a switch from exploitation to exploration. Finally, *β*_*j*_*Controls*_*it*_ is the set of control variables and *ε*_*it*_ is a normally distributed error term.

We estimate *Eq 3* using ordinary least squares (OLS) regression. Before interpreting the results, we first create a baseline model–consisting only of the dependent and control variables–to check for multicollinearity. The variance-inflator test (VIF) shows that the highest score is 1.96 (*International*), which is well within the accepted threshold of 5, and suggests that multicollinearity is not of concern [74].

## 5. Results

### 5.1 Descriptive statistics

Table 1 describes the distribution of motives across acquisitions. It reports, for example, that in 408 cases, the acquirers announced one exploitative motive and zero exploratory motives and that in 416 cases, the acquirers announced one of each motive.

**Table 1:** Motives per acquisition.

		Exploitative Motives				
		0	1	2	3	Total
Exploratory Motives	0	49	408	53	2	512
	1	275	416	82	7	708
	2	67	45	8	0	120
	3	0	3	0	0	3
	Total	391	872	143	9	1,415

Table 2 reports on the acquisitions and the strategy switches. In the columns, we distinguish between acquirers with a ’high’, ’medium’ or ’low’ level of focus, where high implies that the acquirer had an aggregated exploratory strategy for their previous three acquisitions outside the threshold of 30–70%. Medium relaxes this to 35–65% and low relaxes it further to 40–60%. In the rows, we distinguish between a 50%, 40% or 30% switch in strategy, relative to the previous three acquisitions. In other words, Table 2 suggests that there were 102 acquisitions that constitute a large (>50%) shift in strategy, for an acquirer with a highly focused pattern. This number increases to 312 when we relax the size of the shift and/or the level of focus. The numbers between brackets in Table 2 describe the direction of the switch. The first number is the number of switches towards exploration in the focal acquisition, and the second is the number of switches towards exploitation in the focal acquisition. For example, in the case of the 102 acquisitions in the ’High’ and ’50% shift’ cell, we report that there were 52 switches towards exploration and 50 towards exploitation.

**Table 2:** Frequencies per strategy switch (shift to exploratory-exploitative innovation strategy in focal acquisition).

	High (0,30–0,70)	Medium (0,35–0,65)	Low (0,40–0,60)
50% Shift	102 (52–50)	136 (68–68)	174 (83–91)
40% Shift	120 (64–56)	157 (81–76)	196 (97–99)
30% Shift	150 (79–71)	228 (112–116)	312 (145–167)

Finally, Table 3 reports on the min, mean, max, and standard deviation for each of the variables that we employ in our model, as well as the correlations between these. None of the correlations are above 0.7, which is the cut-off used to indicate multicollinearity.

**Table 3:** Descriptive statistics and correlations.

		1	2	3	4	5	6	7	8	9	10	11
1	CAR	1.000										
2	High Focus–Switch 50%	0.042	1.000									
		(0.114)										
3	Prior Performance	0.081	0.030	1.000								
		(0.002)	(0.255)									
4	Geographic Distance	0.023	-0.004	0.076	1.000							
		(0.388)	(0.870)	(0.005)								
5	Relatedness	0.049	-0.016	0.006	-0.033	1.000						
		(0.067)	(0.539)	(0.837)	(0.221)							
6	R&D intensity	-0.037	-0.017	-0.183	-0.040	0.051	1.000					
		(0.161)	(0.515)	(0.000)	(0.130)	(0.054)						
7	International deal	0.050	-0.022	0.105	0.668	0.022	0.009	1.000				
		(0.060)	(0.402)	(0.000)	(0.000)	(0.406)	(0.731)					
8	Motive Count	0.027	-0.020	0.024	0.038	0.075	0.000	0.028	1.000			
		(0.305)	(0.452)	(0.375)	(0.150)	(0.005)	(0.995)	(0.300)				
9	(Log) Acquirer Size	-0.046	0.074	0.195	0.151	-0.235	-0.120	0.181	0.011	1.000		
		(0.088)	(0.005)	(0.000)	(0.000)	(0.000)	(0.000)	(0.000)	(0.673)			
10	(Log) Deal Value	-0.051	0.008	0.156	-0.026	0.113	-0.047	-0.044	0.196	0.336	1.000	
		(0.054)	(0.771)	(0.000)	(0.334)	(0.000)	(0.075)	(0.095)	(0.000)	(0.000)		
11	(Log) Financial Slack	0.065	0.018	0.003	-0.016	-0.023	-0.038	-0.022	-0.015	-0.098	-0.046	1.000
		(0.015)	(0.488)	(0.920)	(0.553)	(0.384)	(0.157)	(0.401)	(0.568)	(0.000)	(0.080)	
	Mean	0.00	0.04	5.57	3536.70	2.07	14.36	0.40	1.29	9.17	5.05	-2.05
	Standard Deviation	0.08	0.19	15.54	3471.91	1.70	34.07	0.49	0.63	1.82	1.67	0.78
	Min	-0.31	0.00	-301.85	0.00	0.00	0.06	0.00	0.00	4.19	2.30	-6.79
	Max	0.52	1.00	58.85	16936.94	4.00	779.78	1.00	4.00	13.04	11.13	-0.02

### 5.2 Hypothesis testing

We test our hypotheses using OLS regressions. Tables 4 and 5 report the results. In each model, we include but do not report industry and year dummies. Table 4 reports the result necessary to test Hypothesis 1. Table 5 reports the result to test Hypothesis 2.

**Table 4:** H1 regression results.

	Main Analysis				Robustness Checking					
	High Focus				Medium Focus			Low Focus		
	(1)	(2)	(3)	(4)	(5)	(6)	(7)	(8)	(9)	(10)
VARIABLES	CAR	CAR	CAR	CAR	CAR	CAR	CAR	CAR	CAR	CAR
Switch 50%		0.020***			0.016***			0.011**		
		(2.827)			(2.761)			(2.074)		
Switch 40%			0.013**			0.011*			0.006	
			(1.986)			(1.936)			(1.199)	
Switch 30%				0.011*			0.012**			0.009**
				(1.881)			(2.485)			(1.997)
(Log) Prior Performance	0.001	0.001	0.001	0.001	0.001	0.001	0.001	0.001	0.001	0.001
	(0.514)	(0.505)	(0.509)	(0.487)	(0.531)	(0.527)	(0.493)	(0.538)	(0.528)	(0.531)
(Log) Firm Size	-0.001	-0.001	-0.001	-0.001	-0.001	-0.001	-0.002	-0.001	-0.001	-0.001
	(-0.720)	(-0.874)	(-0.854)	(-0.863)	(-0.851)	(-0.839)	(-0.914)	(-0.801)	(-0.783)	(-0.851)
(Log) Deal Value	-0.002	-0.002	-0.002	-0.002	-0.002	-0.002	-0.002	-0.002	-0.002	-0.002
	(-0.930)	(-0.979)	(-0.945)	(-0.920)	(-0.975)	(-0.946)	(-0.915)	(-0.964)	(-0.943)	(-0.939)
Method of Payment	-0.000	-0.000	-0.000	-0.000	-0.000	-0.000	-0.000	-0.000	-0.000	-0.001
	(-0.056)	(-0.005)	(-0.020)	(-0.036)	(-0.053)	(-0.052)	(-0.081)	(-0.065)	(-0.060)	(-0.105)
Relatedness	0.002	0.002	0.002	0.002	0.002	0.002	0.002	0.002	0.002	0.002
	(1.476)	(1.440)	(1.447)	(1.476)	(1.415)	(1.434)	(1.484)	(1.478)	(1.479)	(1.522)
International	0.006	0.007	0.007	0.007	0.007	0.007	0.007	0.007	0.007	0.007
	(1.015)	(1.159)	(1.100)	(1.065)	(1.145)	(1.092)	(1.115)	(1.117)	(1.066)	(1.071)
(Log) Potential Slack	0.006**	0.007**	0.006**	0.006**	0.006**	0.006**	0.006**	0.006**	0.006**	0.006**
	(2.340)	(2.355)	(2.335)	(2.315)	(2.302)	(2.304)	(2.252)	(2.319)	(2.321)	(2.288)
(Log) Geographic Distance	-0.000	-0.000	-0.000	-0.000	-0.000	-0.000	-0.000	-0.000	-0.000	-0.000
	(-0.410)	(-0.460)	(-0.426)	(-0.384)	(-0.433)	(-0.408)	(-0.374)	(-0.433)	(-0.411)	(-0.348)
Motives	-0.004	-0.006	-0.005	-0.004	-0.007	-0.005	-0.005	-0.007	-0.005	-0.006
	(-0.342)	(-0.512)	(-0.405)	(-0.323)	(-0.573)	(-0.440)	(-0.413)	(-0.569)	(-0.432)	(-0.478)
Year Dummies	Yes	Yes	Yes	Yes	Yes	Yes	Yes	Yes	Yes	Yes
Industry Dummies	Yes	Yes	Yes	Yes	Yes	Yes	Yes	Yes	Yes	Yes
Constant	-0.023	-0.020	-0.021	-0.021	-0.019	-0.020	-0.019	-0.020	-0.021	-0.019
	(-0.686)	(-0.617)	(-0.647)	(-0.647)	(-0.581)	(-0.621)	(-0.572)	(-0.607)	(-0.646)	(-0.579)
Observations	1,415	1,415	1,415	1,415	1,415	1,415	1,415	1,415	1,415	1,415
Adjusted R-squared	0.031	0.034	0.032	0.032	0.034	0.032	0.033	0.032	0.031	0.032

Robust t-statistics in parentheses.

*** p<0.01

** p<0.05

* p<0.1.

**Table 5:** a. H2 regression results for high focus acquirers. b. H2 regression results for medium and low focus acquirers.

	(11)	(12)	(13)	(14)	(15)	(16)
VARIABLES	CAR	CAR	CAR	CAR	CAR	CAR
Switch to Exploit 50%	0.022*					
	(1.910)					
Switch to Exploit 40%		0.017				
		(1.603)				
Switch to Exploit 30%			0.008			
			(0.904)			
Switch to Explore 50%				0.016**		
				(2.152)		
Switch to Explore 40%					0.008	
					(1.115)	
Switch to Explore 30%						0.012*
						(1.788)
(Log) Prior Performance	0.001	0.001	0.001	0.001	0.001	0.001
	(0.560)	(0.542)	(0.510)	(0.472)	(0.498)	(0.490)
(Log) Firm Size	-0.001	-0.001	-0.001	-0.001	-0.001	-0.001
	(-0.751)	(-0.757)	(-0.743)	(-0.822)	(-0.784)	(-0.841)
(Log) Deal Value	-0.002	-0.002	-0.002	-0.002	-0.002	-0.002
	(-1.017)	(-0.990)	(-0.953)	(-0.904)	(-0.910)	(-0.883)
Method of Payment	-0.001	-0.000	-0.000	0.000	-0.000	-0.000
	(-0.087)	(-0.078)	(-0.072)	(0.009)	(-0.023)	(-0.012)
Relatedness	0.002	0.002	0.002	0.002	0.002	0.002
	(1.448)	(1.446)	(1.476)	(1.466)	(1.471)	(1.474)
International	0.007	0.007	0.006	0.007	0.007	0.007
	(1.088)	(1.050)	(1.014)	(1.077)	(1.048)	(1.066)
(Log) Potential Slack	0.007**	0.007**	0.007**	0.006**	0.006**	0.006**
	(2.401)	(2.382)	(2.357)	(2.305)	(2.316)	(2.288)
(Log) Geographic Distance	-0.000	-0.000	-0.000	-0.000	-0.000	-0.000
	(-0.429)	(-0.421)	(-0.389)	(-0.437)	(-0.414)	(-0.411)
Motives	-0.005	-0.005	-0.004	-0.005	-0.004	-0.003
	(-0.462)	(-0.415)	(-0.357)	(-0.393)	(-0.346)	(-0.298)
Year Dummies	Yes	Yes	Yes	Yes	Yes	Yes
Industry Dummies	Yes	Yes	Yes	Yes	Yes	Yes
Constant	-0.019	-0.020	-0.021	-0.023	-0.023	-0.023
	(-0.591)	(-0.609)	(-0.650)	(-0.698)	(-0.696)	(-0.694)
Observations	1,415	1,415	1,415	1,415	1,415	1,415
Adjusted R-squared	0.033	0.032	0.031	0.032	0.031	0.031
Robust t-statistics in parentheses.						
*** p<0.01						
** p<0.05						
* p<0.1.						

	Medium Focus						Low Focus					
	(1)	(2)	(3)	(4)	(5)	(6)	(7)	(8)	(9)	(10)	(11)	(12)
VARIABLES	CAR	CAR	CAR	CAR	CAR	CAR	CAR	CAR	CAR	CAR	CAR	CAR
Switch to Exploit 50%	0.015						0.007					
	(1.605)						(0.826)					
Switch to Exploit 40%		0.010						0.004				
		(1.193)						(0.482)				
Switch to Exploit 30%			0.007						0.005			
			(1.088)						(0.794)			
Switch to Explore 50%				0.016**						0.015**		
				(2.429)						(2.389)		
Switch to Explore 40%					0.010						0.008	
					(1.547)						(1.284)	
Switch to Explore 30%						0.014**						0.011**
						(2.448)						(2.016)
(Log) Prior Performance	0.001	0.001	0.001	0.001	0.001	0.001	0.001	0.001	0.001	0.001	0.001	0.001
	(0.576)	(0.552)	(0.531)	(0.465)	(0.490)	(0.458)	(0.541)	(0.527)	(0.535)	(0.485)	(0.503)	(0.486)
(Log) Firm Size	-0.001	-0.001	-0.001	-0.001	-0.001	-0.001	-0.001	-0.001	-0.001	-0.001	-0.001	-0.001
	(-0.742)	(-0.743)	(-0.757)	(-0.822)	(-0.803)	(-0.876)	(-0.725)	(-0.726)	(-0.739)	(-0.811)	(-0.784)	(-0.834)
(Log) Deal Value	-0.002	-0.002	-0.002	-0.002	-0.002	-0.002	-0.002	-0.002	-0.002	-0.002	-0.002	-0.002
	(-0.985)	(-0.967)	(-0.943)	(-0.913)	(-0.908)	(-0.886)	(-0.957)	(-0.943)	(-0.952)	(-0.907)	(-0.911)	(-0.890)
Method of Payment	-0.001	-0.001	-0.001	-0.000	-0.000	-0.000	-0.000	-0.000	-0.001	-0.000	-0.000	-0.000
	(-0.095)	(-0.084)	(-0.090)	(-0.011)	(-0.026)	(-0.022)	(-0.080)	(-0.070)	(-0.091)	(-0.013)	(-0.031)	(-0.034)
Relatedness	0.002	0.002	0.002	0.002	0.002	0.002	0.002	0.002	0.002	0.002	0.002	0.002
	(1.455)	(1.457)	(1.480)	(1.438)	(1.455)	(1.477)	(1.488)	(1.480)	(1.487)	(1.447)	(1.467)	(1.502)
International	0.007	0.007	0.006	0.007	0.007	0.007	0.007	0.006	0.006	0.007	0.007	0.007
	(1.060)	(1.031)	(1.026)	(1.091)	(1.066)	(1.107)	(1.045)	(1.026)	(1.010)	(1.079)	(1.054)	(1.093)
(Log) Potential Slack	0.007**	0.007**	0.007**	0.006**	0.006**	0.006**	0.007**	0.007**	0.007**	0.006**	0.006**	0.006**
	(2.380)	(2.366)	(2.349)	(2.257)	(2.280)	(2.222)	(2.358)	(2.349)	(2.350)	(2.268)	(2.293)	(2.255)
(Log) Geographic Distance	-0.000	-0.000	-0.000	-0.000	-0.000	-0.000	-0.000	-0.000	-0.000	-0.000	-0.000	-0.000
	(-0.410)	(-0.406)	(-0.380)	(-0.432)	(-0.412)	(-0.422)	(-0.421)	(-0.414)	(-0.381)	(-0.415)	(-0.402)	(-0.402)
Motives	-0.006	-0.005	-0.005	-0.005	-0.004	-0.004	-0.005	-0.004	-0.005	-0.005	-0.004	-0.005
	(-0.476)	(-0.414)	(-0.384)	(-0.423)	(-0.362)	(-0.343)	(-0.417)	(-0.375)	(-0.393)	(-0.464)	(-0.380)	(-0.385)
Year Dummies	Yes	Yes	Yes	Yes	Yes	Yes	Yes	Yes	Yes	Yes	Yes	Yes
Industry Dummies	Yes	Yes	Yes	Yes	Yes	Yes	Yes	Yes	Yes	Yes	Yes	Yes
Constant	-0.020	-0.021	-0.021	-0.022	-0.023	-0.022	-0.021	-0.022	-0.021	-0.022	-0.023	-0.022
	(-0.600)	(-0.622)	(-0.626)	(-0.676)	(-0.686)	(-0.663)	(-0.644)	(-0.661)	(-0.636)	(-0.675)	(-0.686)	(-0.669)
Observations	1,415	1,415	1,415	1,415	1,415	1,415	1,415	1,415	1,415	1,415	1,415	1,415
Adjusted R-squared	0.032	0.031	0.031	0.032	0.031	0.032	0.031	0.030	0.031	0.032	0.031	0.032
Robust t-statistics in parentheses.												
*** p<0.01												
** p<0.05												
* p<0.1.												

#### 5.2.1 Hypothesis one: The effect of switching

Table 4 presents ten regressions to test Hypothesis 1. Model 1 reports the effect of the control variables on market reaction and, as such, acts as the statistical basis. This model presents results based on 1389 observations. With an R-squared = 0,031, the explanatory power of Model 1 is in line with, or better than, other event-based studies (e.g. [27]).

Models 2–4 consider the effect of a switch when the acquirer has high focus; that is, an acquirer with an aggregated exploratory strategy outside the threshold of 30–70%. Model 2 reports that a switch in strategy of at least 50% has a positive and significant effect on market reactions. The Switch coefficient is positive and significant at the 1% level. Models 3 and 4 suggest that this effect is also present for less pronounced switches of 40%, significant at the 5% level and 30%, significant at the 10% level.

Models 5–10 repeat the exercise for robustness checking purposes. Models 5–7 consider the effect of a switch when the acquirer has ’medium’ focus; that is, when the acquirer has an aggregated exploratory strategy outside the threshold of 35–65%. As in the case of high focus acquirers, Model 5 reports that a switch in strategy, of at least 50%, has a positive and significant effect on market reactions, and Model 6 reports a significant effect for a 40% switch. Interestingly, Model 7 reports a positive effect of a 40% switch. In each case, the Switch coefficient is positive and significant at the 10% level. Similarly, Models 8–10 consider the effect of a switch when the acquirer has low focus; that is, when the acquirer has an aggregated exploratory strategy outside the threshold of 40–60% threshold. Model 8 shows a significant effect for 40% switches and Model 10 reports a significant effect for 30% switches, both at the 5% level. Model 9 does display an insignificant effect for 40% switches.

#### 5.2.2 Hypothesis two: The direction of the switch

Table 5A presents six regressions to test Hypothesis 2 for high focus acquirers. Model 11 reports that a 50% switch to exploit has a positive and significant effect on the market reaction. Model 12 and 13 report the effect for a 40% and 30% switch to exploit, which both yielded positive yet insignificant effects. Model 14–16 show the results for switches to exploration. We like to inform the reader that for this subgroup, we only had 52 cases. Model 14 reports a positive and significant effect on market reactions for 50% switches. Model 15 showed a positive and insignificant effect for 40% switches to explore, and Model 16 reports a positive and significant effect for 30% switches to explore.

Table 5B reports the results for acquirers with medium and low focus. For medium-focused acquirers, 50% and 30% switches to exploration lead to positive and significant results (see Model 4 and 6). All other models for acquirers with a medium focus display positive and insignificant results. Also, for low-focused acquirers, 50% and 30% switches to exploration yield positive and significant results (see Model 10 and 12). Similar to the models for acquirers with a medium focus, all other models report positive and insignificant results.

## 6. Discussion

Our results suggest that the market takes a portfolio perspective when reacting to an acquisition. In support of the ambidexterity literature, which says that the firm should both explore and exploit, we show that the market responds positively to a switch from one type of strategy to another. More precisely, we find that the market reacts positively to a switch towards exploration after a series of exploitation and to a switch towards exploitation after a series of exploration. In either case, we suggest that the switch in strategy is interpreted by the market as a value-creating step towards building a more balanced position. Then, zooming in on the direction of the shift, we find that the market responds more positively to a switch towards exploration after a series of exploitation, compared with the alternative. This is the case, we suggest, because a switch to exploration signals that the firm is ready to engage in high-quality innovation, aimed at long-term performance and, after a series of exploitative acquisitions, it has the slack resources necessary to fund these innovations too. Considering the underlying data we used to derive acquisition motives, we also showed that press releases’ content, beyond numeric information, is incorporated by investors in deal evaluations.

## 7. Conclusion

### 7.1 Practical implications

Our findings have a number of clear implications for managers. First, and at the highest level, our results warn the manager to be aware of the signals that they send. Previous work suggeststhat markets are sensitive to even the smallest signal regarding the likely performance of an acquisition. We show that the market even takes the motives underlying the firm’s previous acquisitions into account too, when reacting to the focal acquisition. Second, our results highlight the importance of maintaining a balanced acquisition portfolio. Exploitation cuts costs, creates slack, improves the firm’s short-term financials, and it is low risk. Exploration, by contrast, is an uncertain, high-risk ’leap into the unknown’. It is easy to see, therefore, why markets and managers might prefer exploitation over exploration. However, exploration leads to the sorts of breakthrough innovations that are necessary to secure long-term performance. It is important, therefore, to explore and exploit in equal measures. Our results suggest not only that the market is aware of this, but that it rewards managers who switch strategy, back and forth between exploration and exploitation, in order to build a balanced firm. Finally, our results suggest that the market rewards explorative acquisitions, with the important caveat that they are announced at the end of a series of exploitative acquisitions. Exploration is high-risk and expensive, but it is also critical to creating new revenue streams. Our findings suggest that the market reacts positively to a switch to exploration, which signals that the firm is ready to engage in high-quality innovation, once it has completed a series of exploitative acquisitions to build the slack necessary to fund it.

### 7.2 Theoretical implications

Our findings contribute to a number of academic discussions. Our theoretical implications allow us to extend the discussion on [30] framework by applying it to the topic of serial acquisitions, as well as to contribute to the discussion on signaling theory by introducing the switch in strategy as another relevant signal. Our main contributions, however, are to the literature on mergers and acquisition, on the one hand, and to the discussion of ambidexterity, on the other.

Principally, we contribute to the literature on acquisitions (e.g., [6,75]) in a number of ways. First, we contribute to the general discussion of acquisition performance by showing that the market takes a portfolio perspective of the firm’s acquisitions when reacting to the focal acquisition. This is an important contribution, given the rather mixed evidence that presently exists regarding acquisitions (e.g., [76,77]). Second, we contribute to work on acquisition motives (e.g., [2,24]), by showing that the market takes the motive behind the acquirer’s previous acquisitions into account when reacting to the focal acquisition. This is important given the limited attention that has been dedicated to acquisition motives. Third, we contribute to the work on serial acquirers by incorporating an acquirer’s previous acquisition strategy in explaining firm performance in response to calls to do so (e.g. [6,16,17]). Fourth, we contribute to the discussion on target selection by showing that while a switch towards exploitation after a series of exploration does not provoke a market reaction, a switch towards exploration after exploitation does. This is an interesting finding, given that the literature suggests that–when treated in isolation–the market prefers immediate and low-risk exploitation over distant and high-risk exploration (e.g. [2]).

Beyond our contribution to the literature on acquisitions, additionally, we make a number of contributions to the literature on ambidexterity. For instance, [2] find that the market reacts positively to the announcement of exploitation-based acquisitions, negatively to the announcement of exploitation-based acquisitions to exploration, and most negatively to the announcement of ambidextrous acquisitions which seek to simultaneously explore and exploit. From this, one could easily conclude, as the authors do, that the market only supports exploitative acquisitions. Our findings add further depth to this discussion as we investigated the market’s reactions to serial acquirers that switch strategy. Being the first to the best of our knowledge to centre in on the direction of the shift, our findings challenge conventional wisdom in M&A research that assumes a relative stability in merger motives. We show, first, that the market supports ambidexterity and that it favors to what we alude to as ‘moving motives’, a motive portfolio that is oscillating back and forth between periods of exploitation and exploration. Second, we show that the market welcomes a switch to exploration, in particular when the switch is made after a series of exploitive acquisitions. In so doing, we provide insights on the role and importance of ambidexterity in acquisitions, while also providing insight on the way in which ambidexterity can be attained through acquisitions.

### 7.3 Limitations and future direction

This research provides a first step in understanding investor reactions to changes in the strategy of serial acquirers. Like all research, it is subject to a number of limitations which, in turn, can be a source of future research. In this section, we highlight five limitations.

First, we test our hypotheses using a sample of acquisitions from four high-tech industries. We do so, arguing that the high volume of acquisitions and the high knowledge burden that they place on the investor makes the industry an attractive setting. We hope that future research will explore the generalizability of our findings outside these industries.

Second, we make use of an event study to describe market responses. We recognize that assuming that markets can correctly incorporate new information to update firm value has been challenged (e.g., [78]) in finance (e.g., [79]) and strategy (e.g., [80]). Abnormal returns are not only influenced by cognitive biases [81], but also by other factors that affect mood, such as good weather [82] and bad news[83]. We hope future research will explore the relationship we uncovered using other measures.

Thirdly, we assume that all press releases are the same honest signals of true intent. We make this assumption because, in the U.S., for example, it is a criminal offense to mislead the market. However, the reality is that managerial motives play a role in acquisition (e.g. [84]), and there are significant differences in the content of the announcements. Managers are also known to engage in ’impression offsetting’ and to release information to offset the market’s negative reaction [85,86]. Research shows that the tone of the message (e.g., [87]), as well as the reputation of the sender (e.g., [88]), matter. We hope future research will explore how the type and tone of the message affects the relationship we present.

Fourth, we describe serial acquirers as firms that make three acquisitions in ten years. However, the frequency of acquisitions between acquirers varies significantly, from 56 acquisitions in ten years, on the one end, to four at the other. Various scholars (e.g. [41,89]) argue that the time between acquisitions can significantly influence firm performance, as do firm-level characteristics, such as the size of the integration teams, their level of experiences [90–92]. We ignore this possibility and treat all serial acquirers as the same. We hope that future research will enrich our analysis by adding these variables to the analysis.

Lastly, surprisingly, our results suggest that investors do not react significantly to a switch to exploitation. These acquisitions are relatively low risk and have a high probability of increasing performance [58]. The fact that no effect is measured could imply that investors do not perceive the switch to be significant after completing several potentially high-risk and highly rewarding exploratory acquisitions. We invite future research to unravel the underlying mechanisms to such market behavioral responses.

### 7.4 Final conclusions

In this paper, we investigate the market’s reactions to serial acquirers that switch strategy. We collect data on 204 serial acquirers in four high-tech industries, and used March’s (1991) explore-exploit framework, to classify these firms’ acquisitions. We distinguish, for example, between exploration-based acquisitions, conducted after a series of exploitation-based acquisitions, and visa versa. Our results suggest that the market takes a portfolio perspective when reacting to an acquisition. In support of the ambidexterity literature, which suggests that the firm should explore and exploit in equal measure, we show that the market responds positively to a switch from one type of strategy to another. Then, zooming in on the direction of the shift, we find that the market responds more positively to a switch towards exploration after exploitation, compared with the alternative. We argue that this is the case because in an innovation context, for example, the switch to exploration signals that the firm is ready to engage in radical innovation, aimed at long term performance and, after a series of exploitative acquisitions, it has the slack resources necessary to fund these innovations too. In so doing, we contribute to the literature on acquisition motives by showing that prior announcements matter in explaining market reactions, and we contribute to the literature on ambidexterity by showing that the market favours firms that oscillate between exploration and exploitation.

## Supporting information

S1 File Anon sample. (DTA)
